# Impact of left-heart myopathy on mitral valve stenosis assessment and interventional outcomes: an *in-silico* trial

**DOI:** 10.1093/ehjdh/ztaf097

**Published:** 2025-08-19

**Authors:** Gitte P H van den Acker, Sebastiaan Dhont, Tim van Loon, Timothy W Churchill, Frank Timmermans, Tammo Delhaas, Philippe B Bertrand, Joost Lumens

**Affiliations:** Department of Biomedical Engineering, CARIM Cardiovascular Research Institute Maastricht, Maastricht University, Universiteitssingel 40, Maastricht 6229, The Netherlands; Department of Biomedical Engineering, CARIM Cardiovascular Research Institute Maastricht, Maastricht University, Universiteitssingel 40, Maastricht 6229, The Netherlands; Faculty of Medicine and Life Sciences, LCRC, Hasselt University, Diepenbeek, Belgium; Department of Cardiology and Future Health, Ziekenhuis Oost-Limburg, Genk, Belgium; Department of Biomedical Engineering, CARIM Cardiovascular Research Institute Maastricht, Maastricht University, Universiteitssingel 40, Maastricht 6229, The Netherlands; Cardiovascular Research Center, Massachusetts General Hospital, Boston, MA, USA; Department of Cardiology, University Hospital Ghent, Ghent, Belgium; Department of Biomedical Engineering, CARIM Cardiovascular Research Institute Maastricht, Maastricht University, Universiteitssingel 40, Maastricht 6229, The Netherlands; Faculty of Medicine and Life Sciences, LCRC, Hasselt University, Diepenbeek, Belgium; Department of Cardiology and Future Health, Ziekenhuis Oost-Limburg, Genk, Belgium; Department of Biomedical Engineering, CARIM Cardiovascular Research Institute Maastricht, Maastricht University, Universiteitssingel 40, Maastricht 6229, The Netherlands

**Keywords:** Mitral valve stenosis, CircAdapt, Computational modelling and simulation, Echocardiography, *In-silico* clinical trial

## Abstract

**Aims:**

The shift in mitral stenosis (MS) aetiology from rheumatic to calcific valve disease complicates distinguishing valve-related from myocardial-driven haemodynamic abnormalities. This study examines how left-heart myopathy influences flow velocity-based echocardiographic MS severity assessment and evaluates haemodynamic changes following mitral valve (MV) intervention at rest and during exercise.

**Methods and results:**

The CircAdapt biophysical model was used to create a virtual cohort with varying MS severity, left ventricular (LV) compliance, and left atrial (LA) function. Mean gradient (MG) was evaluated alongside left-heart pressures at rest and during exercise. To study acute haemodynamic effects of MV intervention, the mitral valve’s effective orifice area was restored to 5.9 cm². MG showed variation of 1 mmHg attributable to left-heart myopathy. Following virtual MV intervention for clinically significant MS, mean left atrial pressure (mLAP) decreased by 50% in patients with preserved myocardial function but remained elevated in those with LV and LA dysfunction due to persistently elevated LV end-diastolic pressure, resulting in persistently impaired exercise capacity.

**Conclusion:**

Virtual patient cohorts suggest that MV intervention reduces MG but may not normalize mLAP in patients with impaired LV and LA function. Persistent myocardial dysfunction may limit both symptomatic and exercise capacity improvement, despite successful intervention. As percutaneous treatment options expand, distinguishing myocardial from valve-driven abnormalities is essential for accurate assessment, patient selection, and optimizing outcomes.

## Graphical Abstract

**Graphical Abstract:**
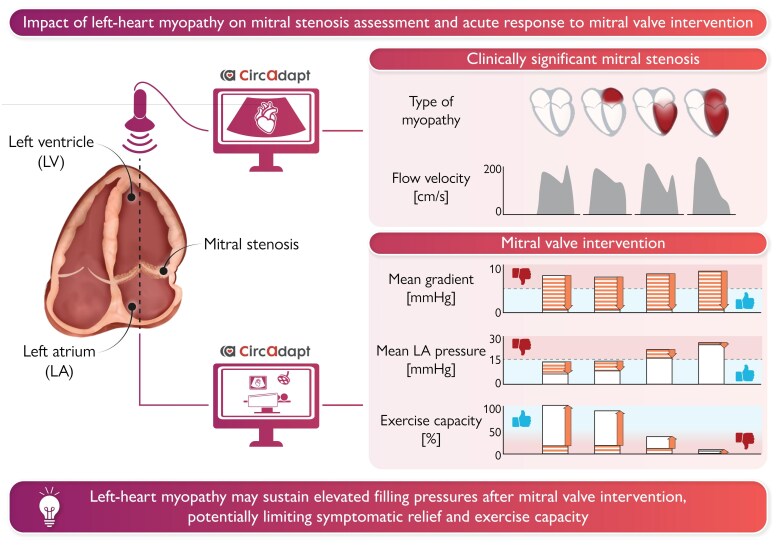
Impact of left-heart myopathy on echocardiographic diagnostics for mitral valve stenosis severity and acute response to mitral valve intervention at rest and during exercise.

## Introduction

**Figure 1:**
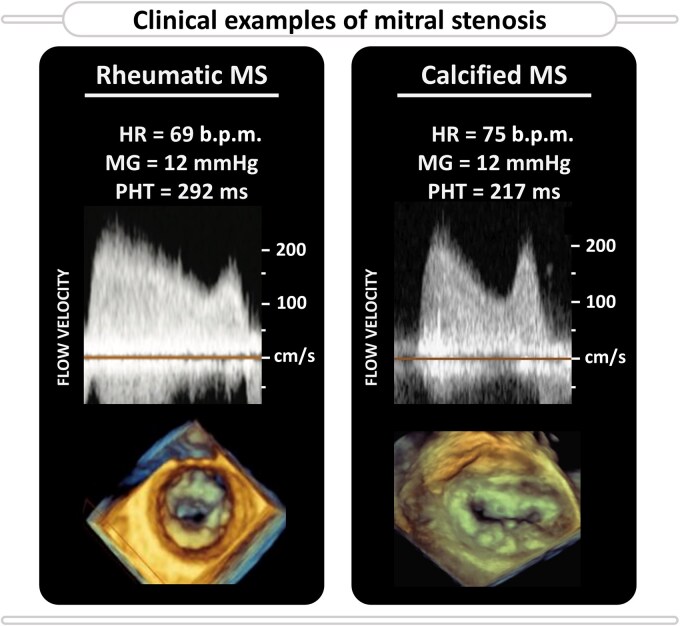
Clinical examples of the mitral flow velocity profiles and valve anatomical features of a rheumatic mitral stenosis (MS) (left panel) vs. calcified MS (right panel). HR, heart rate; MG, mean gradient; PHT, pressure half-time.

Mitral valve stenosis (MS) is characterized by the narrowing of the mitral valve (MV) orifice, obstructing blood flow from the left atrium (LA) into the left ventricle (LV), thereby increasing LA pressure. If untreated, chronic elevation of LA pressures can lead to progressive structural and functional changes in the atrium, often culminating in complications such as atrial fibrillation, pulmonary hypertension, and ultimately congestive heart failure.^1,2^ In recent decades, the clinical profile of MS patients, particularly in developed countries, has shifted. The predominant aetiology has evolved from isolated rheumatic valve disease among otherwise healthy individuals to degenerative calcified valve disease in frail patients with various comorbidities, such as concomitant myocardial, valvular, pulmonary, and vascular diseases (*Figure 1*).^3–6^ This evolution presents new challenges for diagnosis as well as for interventional considerations.

Transthoracic echocardiography is the primary tool for assessing MS severity, with treatment decisions guided by the integration of patient symptoms with echocardiographic indices and anatomical appearance of the valve.^7^ However, the interpretation of velocity-based indices such as mean gradient (MG) and pressure half-time (PHT) has become increasingly complex in the context of concurrent myocardial disease, as these indices are flow-dependent and may be confounded by concomitant myocardial disease, mitral regurgitation, and variations in loading conditions.^6,7^ This raises critical questions about the accuracy of severity grading and, importantly, the expected benefits of MV intervention in patients with complex cardiac comorbidities.

As percutaneous MV interventions become more widely available, there is a growing need to better identify patients that will experience symptomatic and haemodynamic improvement following intervention. This is particularly important as interventions in patients with myocardial dysfunction may result in sub-optimal outcomes despite technically successful valve procedures.^7^

Biophysical computational models of the human cardiovascular system offer unique insights into the complex interplay between the atrium, valve, and ventricle by enabling the study of abnormal valvular and myocardial characteristics in a controlled *in-silico* environment. These models are particularly valuable for examining not only resting haemodynamics but also exercise responses, a critical aspect of patient symptoms and quality of life that is difficult to systematically study in clinical settings. In this study, we performed an *in-silico* clinical trial to explore two important aspects of managing patients with MS: (i) evaluating the accuracy of flow velocity-based echocardiographic indices for grading MS severity in the setting of left-heart myopathy, and (ii) predicting the acute haemodynamic response to MV intervention both at rest and during exercise, with particular focus on how underlying myocardial characteristics may limit functional improvement despite successful valve intervention.

## Methods

### Model description

**Figure 2:**
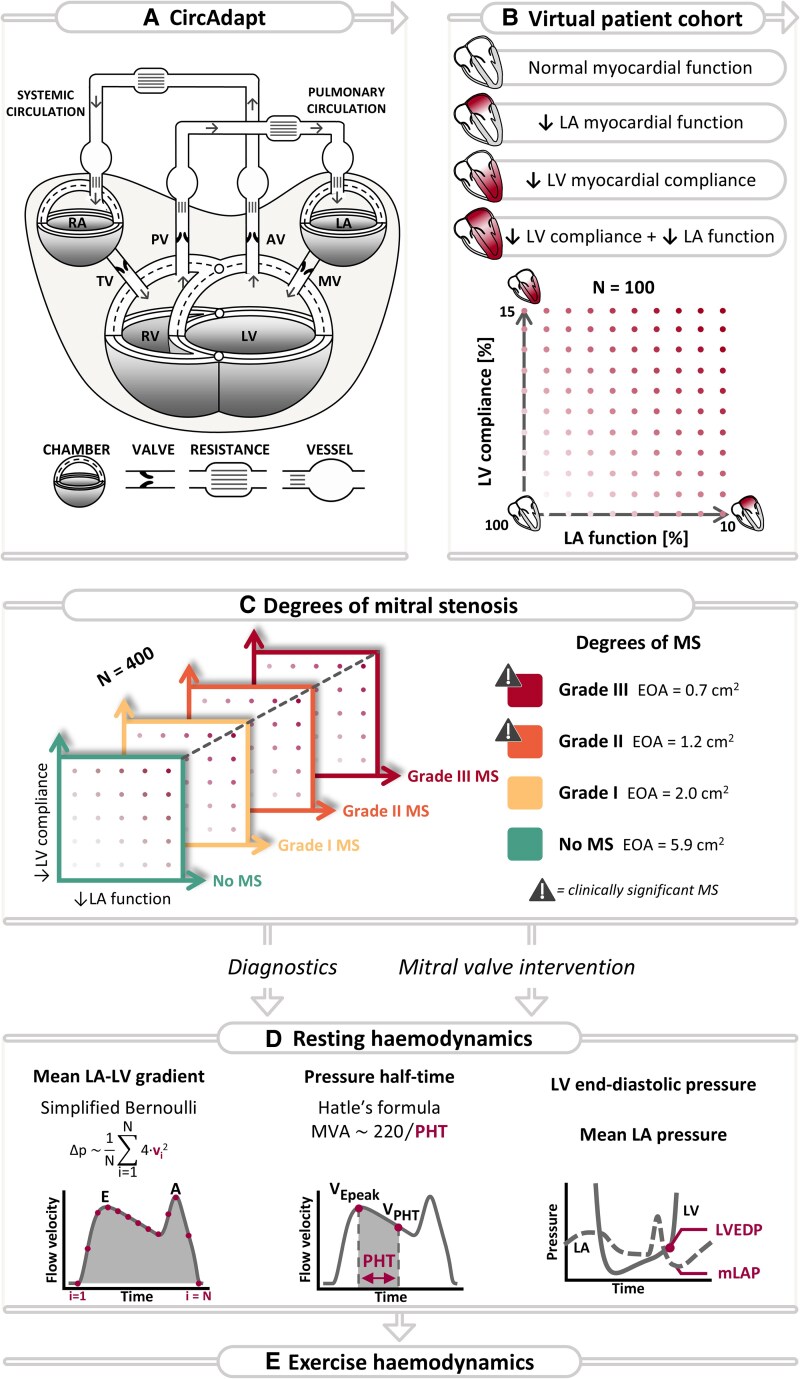
Schematic representation of the methods used in the current study. (*A*) Structure of the closed-loop CircAdapt computational model. (*B*) The virtual patient cohort with varying degrees of left ventricular (LV) and/or left atrial (LA) (dys)function. (*C*) For each virtual patient, four increasing degrees of mitral stenosis (MS) were simulated. (*D*) The resulting haemodynamics were used to compute the non-invasively obtained mean gradient and pressure half-time. LV end-diastolic pressure and mean LA pressure were determined from pressure traces to quantify the acute effects of mitral valve (MV) intervention. (*E*) Evaluation of exercise haemodynamics. AV, aortic valve; EOA, effective orifice area; LA, left atrium; LV, left ventricle; MVA, mitral valve area; p, pressure; PV, pulmonary valve; RA, right atrium; RV, right ventricle; TV, tricuspid valve; v, flow velocity.

The closed-loop CircAdapt biophysical model of the human cardiovascular system was used to simulate cardiovascular mechanics and haemodynamics in virtual patients with varying degrees and combinations of MS severity and left-heart myopathy (www.circadapt.org). CircAdapt enables beat-to-beat simulations of physiological signals in healthy and diseased conditions through modules representing cardiac chambers, valves, major blood vessels, peripheral vasculature, and the pericardium (*Figure 2A*). Details on the model have previously been described.^8–10^ Using well-established biophysical and physiological principles, ordinary differential equations are formulated and solved, enabling fast and realistic simulations of cardiovascular mechanics and haemodynamics over a wide range of (patho-) physiological conditions.

In CircAdapt, the MV is represented as a dynamic orifice whose area varies over time throughout the cardiac cycle depending on the pressure gradient. During diastole, blood transit causes a pressure decrease attributable to inertia and Bernoulli effects, assuming unsteady, incompressible, and non-viscous plug flow (see *Supplementary material A*). The effective mitral orifice areas (EOAs) prescribed to the model serve as the ground truth measures of MS severity. The EOA was initially set to 5.9 cm^2^ for a competent MV.^11^

### Simulation of virtual patient cohorts

To assess the haemodynamic impact of MS in the presence of various degrees of left-heart myopathy, a cohort of 100 virtual patients with different combinations of impaired LA function and reduced LV compliance was generated (*Figure 2B*). The myocardial characteristics range from healthy myocardium, typical in rheumatic MS, to complex myocardial abnormalities affecting both LV compliance and LA function, characteristics of heart failure with preserved ejection fraction (HFpEF), thus reflecting the heterogeneous, comorbid population commonly observed in degenerative MV disease.^6^ These virtual patients were categorized into four sub-cohorts: (i) healthy myocardial tissue, (ii) impaired LA myocardial function, (iii) reduced LV myocardial compliance, and (iv) reduced LV compliance combined with impaired LA function.

### Simulation of impaired LA function

For impaired LA function (cohort 2), LA myocardial contractility was gradually reduced in 10 equal steps until an LA volume index (LAVI) > 34 mL/m^2^ was obtained,^12,13^ assuming a body surface area of 1.88 m^2^.^14^ This resulted in a maximum decrease of the LA active stress component by 90%, simulating reduced atrial contractile function while keeping intrinsic myocardial stiffness constant. As a result, LA maximum volume increased to 72 mL with an LA total ejection fraction (EF) to 42%. The resulting volume overload led to LA dilatation, which consequently raised LA operational chamber stiffness during the passive reservoir phase, despite unaltered intrinsic myocardial stiffness properties. LA operational chamber stiffness,^15^ defined as the slope of the linear line connecting the minimum and maximum LA pressure points during the reservoir phase with corresponding volumes, increased from 0.27 to 0.35 mmHg/mL (see *Supplementary material D*).^16^ LV end-diastolic volume (LVEDV) and LV EF were 145 mL and 50%, respectively.

### Simulation of reduced LV compliance

For reduced LV compliance (cohort 3), the LV passive stress component was increased in 10 equal steps until both LV end-diastolic pressure (LVEDP) and mean LA pressure (mLAP) exceeded the elevated filling pressure threshold. End-diastole was identified at MV closure. An LVEDP > 16 mmHg or mLAP > 15 mmHg was considered pathological.^13^ This resulted in a maximum increase of the intrinsic passive stress component by 85%, raising LVEDP from 10 to 27 mmHg and mLAP from 7 to 16 mmHg in the absence of any MS. LV end-diastolic elastance (Eed), derived from the tangent of the end-diastolic pressure–volume relation (see *Supplementary material E*),^17^ increased from 0.16 to 0.74 mmHg/mL. The resulting LVEDV was 143 mL and LV EF 50%.

Finally, the above-described myocardial tissue alterations for the LV and LA were combined (cohort 4), leading to an LV Eed of 0.77 mmHg/mL, LA maximum volume of 102 mL, LA total EF of 32%, and LA operational stiffness of 0.56 mmHg/mL. LVEDV and LV EF were 141 mL and 51%, respectively. These values reflect the most severe case of combined LV and LA dysfunction, with all LA measurements consistent with reported ranges for LA function in patients with HFpEF.^16^

All virtual patients were subjected to constant haemodynamic parameters, with cardiac output (CO) maintained at 5.1 L/min, mean arterial pressure (MAP) at 92 mmHg, and heart rate (HR) at 70 beats per minute, consistent with previous studies^9^ and typical resting conditions. These haemodynamic parameters were maintained using a pressure-flow control module, simulating homeostatic regulation by adjusting systemic vascular resistance and circulating blood volume. This allowed for the assessment of the isolated effects of MS and left-heart myopathy, independent of flow variability. To address potential variations in HR and transmitral flow, as commonly observed in clinical scenarios involving tachycardia or concomitant mitral regurgitation, sensitivity analyses were performed where the virtual cohorts were subjected to changes in preload and HR.

### Simulation of mitral stenosis

To create virtual patient cohorts with varying degrees of MS severity, the simulated MV EOA was reduced from 5.9 cm^2^ (initial MV EOA) to 2.0, 1.2, and 0.7 cm^2^, referred to in this study as Grades I, II, and III MS, respectively (*Figure 2C*). According to guidelines, Grades II and III MS represent clinically significant MS.^7^ These MS severities were prescribed to the virtual cohort (*Figure 2C*), creating 400 virtual patients with varying degrees of MS severity and left-heart myopathy. All virtual patients were subjected to preserved flow- and pressure conditions using the above-described control module, regardless of myocardial characteristics or MS severity.

### Quantification of mitral stenosis severity at rest

Two flow velocity-based echocardiographic indices for MS classification were computed: MG and PHT (*Figure 2D*). MG was calculated using the simplified Bernoulli equation (*Figure 2D*) on the instantaneous diastolic flow velocities across the MV (see *Supplementary material B*). PHT was calculated as the time taken for the passive diastolic (*E*) peak flow velocity to decay to $v_{\mathrm{PHT}}$, defined as $v_{\mathrm{PHT}}=\frac{v_{\mathrm{MAX}}}{\surd 2}$ (*Figure 2D*; *Supplementary material C*), from which mitral valve area (MVA) was derived using an empirical formula introduced by Hatle *et al*.^18^ In addition to the echocardiographic indices, LVEDP and mLAP were determined from the LV and LA pressure traces, respectively.

### Simulation and quantification of mitral valve intervention at rest

To simulate MV intervention and evaluate its acute haemodynamic effects, MV EOA was restored to its initial value of 5.9 cm^2^ from each MS severity state. LVEDP and mLAP were then reassessed to quantify the haemodynamic improvement following MV intervention at rest.

### Simulation of virtual exercise haemodynamics

To evaluate the determinants of exercise intolerance in MS and left-heart myopathy before and after intervention, and to explore potential mechanisms behind sub-optimal response, a virtual exercise protocol was conducted pre- and post-valve intervention (*Figure 2E*). As previously published,^11^ exercise was simulated by simultaneously increasing CO and HR through the pressure-flow control module, assuming a linear CO-HR relationship, while maintaining a constant MAP. This approach allows assessment of the cardiovascular system’s ability to meet circulatory demand.

Under the assumption that elevated mLAP is a main limiting factor of exercise tolerance, exercise intensity was progressively increased from rest until mLAP exceeded 25 mmHg. This threshold represents the physiological limitation imposed by pulmonary congestion, caused by the retrograde transmission of elevated left-sided filling pressures into the pulmonary circulation, and aligns with post-capillary wedge pressures reported in HFpEF patients during exercise.^19^

To enable quantitative comparisons across myocardial characteristics, CO_max-pred_ was defined as the predicted maximum CO at the 25 mmHg mLAP threshold, serving as a surrogate for peak exercise capacity. This was normalized against a healthy reference simulation (no left-heart myopathy, no MS), representing 100% predicted exercise capacity. Additionally, the relationship between mLAP and CO was linearly approximated to quantify the mLAP/CO slope as an index of the haemodynamic load imposed on the pulmonary circulation during exercise.

## Results

### Effect of isolated mitral stenosis on cardiac haemodynamics

**Figure 3:**
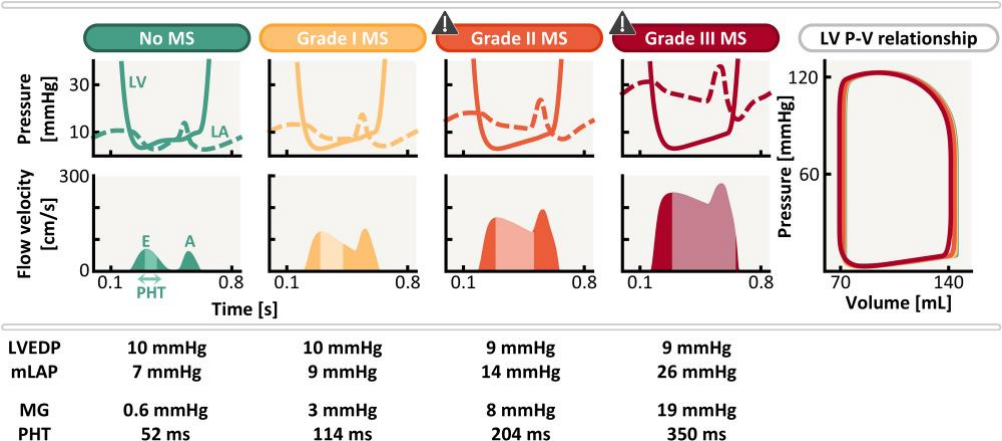
The effect of increasing mitral stenosis (MS) severity on left ventricular (LV) and left atrial (LA) pressure (top row), mitral inflow velocity (bottom row), pressure–volume relations (right column), mean gradient (MG) and pressure half-time (PHT). A, late diastolic inflow velocity; E, early diastolic inflow velocity; mLAP, mean left atrial pressure; LVEDP, left ventricular end-diastolic pressure; P–V, pressure–volume.

In the healthy myocardium cohort, pressure tracings (*Figure 3*, top) demonstrate increasing transmitral pressure differences with increasing MS severity, raising MG from 0.6 mmHg without MS to 19 mmHg for Grade III MS. Despite increasing MS severity, LVEDP remains constant at 9–10 mmHg, while mLAP progressively rises from 7 to 26 mmHg. The presence of Grade II MS (MV EOA = 1.2 cm^2^) leads to mLAP approaching the pathological threshold for elevated filling pressure. The LV inflow patterns (*Figure 3*, bottom) reveal that the presence of MS enhances transmitral flow velocities and prolongs E-wave deceleration, increasing PHT from 52 to 114 ms in Grade I MS and up to 350 ms in Grade III MS.

### Effect of myocardial disease on mitral stenosis classification

**Figure 4:**
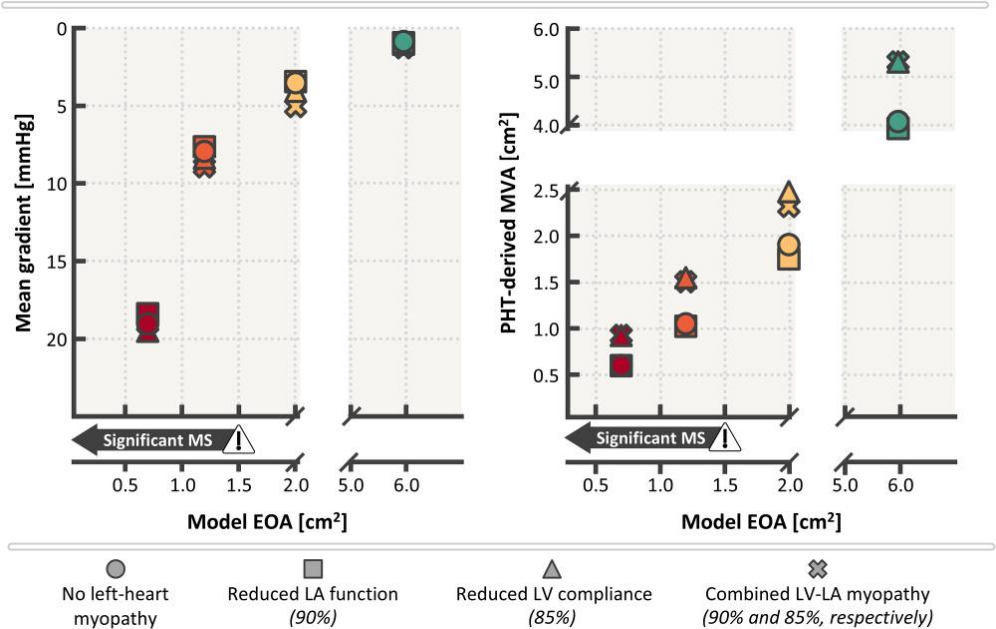
Influence of left-heart myopathy on echocardiographic mitral stenosis (MS) classification using mean gradient (MG, left panel) and pressure half-time (PHT, right panel). EOA, effective orifice area; LA, left atrium; LV, left ventricle; MV, mitral valve; MVA, mitral valve area.

**Figure 5:**
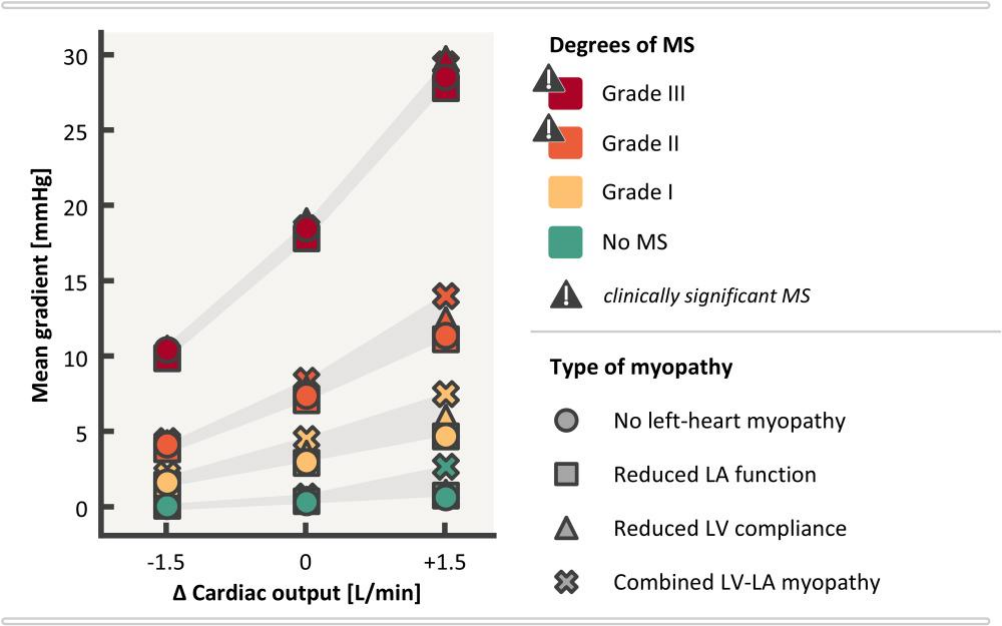
Classification of mitral stenosis (MS) severity based on mean gradient for various cardiac outputs and left-heart myopathies. LA, left atrium; LV, left ventricle.

**Figure 6:**
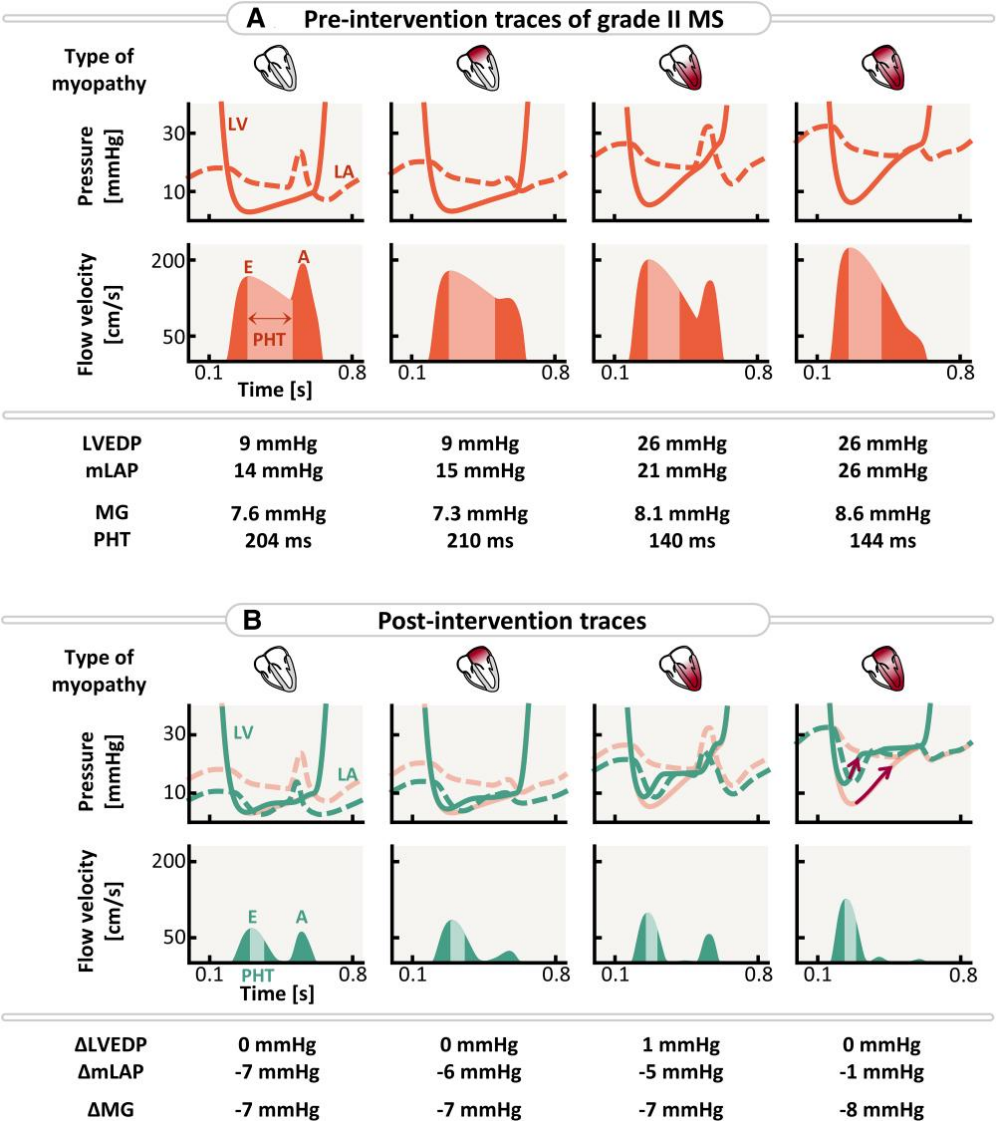
Pressure traces, transmitral flow profiles, and (non-)invasive indices for four virtual patients at the extremes of the myocardial spectrum, both pre- and post-mitral valve intervention. A, late diastolic inflow velocity; E, early diastolic inflow velocity; MG, mean gradient; mLAP, mean left atrial pressure; MS, mitral stenosis; LA, left atrium; LV, left ventricle; LVEDP, left ventricular end-diastolic pressure; PHT, pressure half-time.

*Figure 4* demonstrates MS classification in virtual patients at the extremes of the myocardial spectrum. A non-linear increase is observed in MG as MV EOA decreases. MG is generally below 5 mmHg for Grade I MS or less, ranges from 5 to 10 mmHg for Grade II MS and exceeds 10 mmHg for Grade III MS. Notably, the MG values for each virtual patient exhibit variability at a given MV EOA, especially in cases involving reduced LV compliance. MG ranges from 3.2 to 4.7 mmHg in Grade I MS, from 7.3 to 8.6 mmHg in Grade II MS (corresponding flow velocity profiles shown in *Figure 6B*), and from 18.1 to 19.2 mmHg in Grade III MS. *Figure 5* further illustrates that this variability is amplified with increased transmitral flow, such as in the presence of mitral regurgitation, while reduced transmitral flow tends to decrease MG variability. Additional analyses where virtual patients were subjected to changes in HR are reported in *Supplementary material F.*

The virtual patients demonstrate a discrepancy between MV EOA and PHT-derived MVA in the presence of abnormal myocardial characteristics. In the absence of MS, MV EOA was fixed at 5.9 cm^2^, while the PHT-derived MVA varies from 4.0 to 5.5 cm^2^. MVA was estimated between 1.8 and 2.5 cm^2^ for an MV EOA of 2.0 cm^2^, 1.0 to 1.6 cm^2^ for an MV EOA of 1.2 cm^2^, and 0.6 to 0.9 cm^2^ for an MV EOA of 0.7 cm^2^. Moreover, the presence of reduced LV compliance led to an overestimation of PHT-derived MVA. Additionally, MVA estimation becomes less reliant on LV compliance as MV EOA decreases, with a smaller range observed for Grade III MS compared with Grade II MS.

### Acute haemodynamic effects of mitral valve intervention at rest

*Figure 6* demonstrates that restoring the MV EOA to its initial value of 5.9 cm^2^ through MV intervention reduces MG by 7 mmHg in cases with no left-heart myopathy, impaired LA function and reduced LV compliance, and by 8 mmHg in combined LV and LA dysfunction. While mLAP decreases across all virtual patients, the reduction is minimal, only 1 mmHg, in the presence of combined LV and LA myopathy. The removal of Grade II MS does not markedly affect LVEDP, regardless of myopathy type. Instead, the intervention allows LV pressure to quickly equilibrate with LA pressure (red arrows), while LVEDP is primarily influenced by LV compliance.

**Figure 7:**
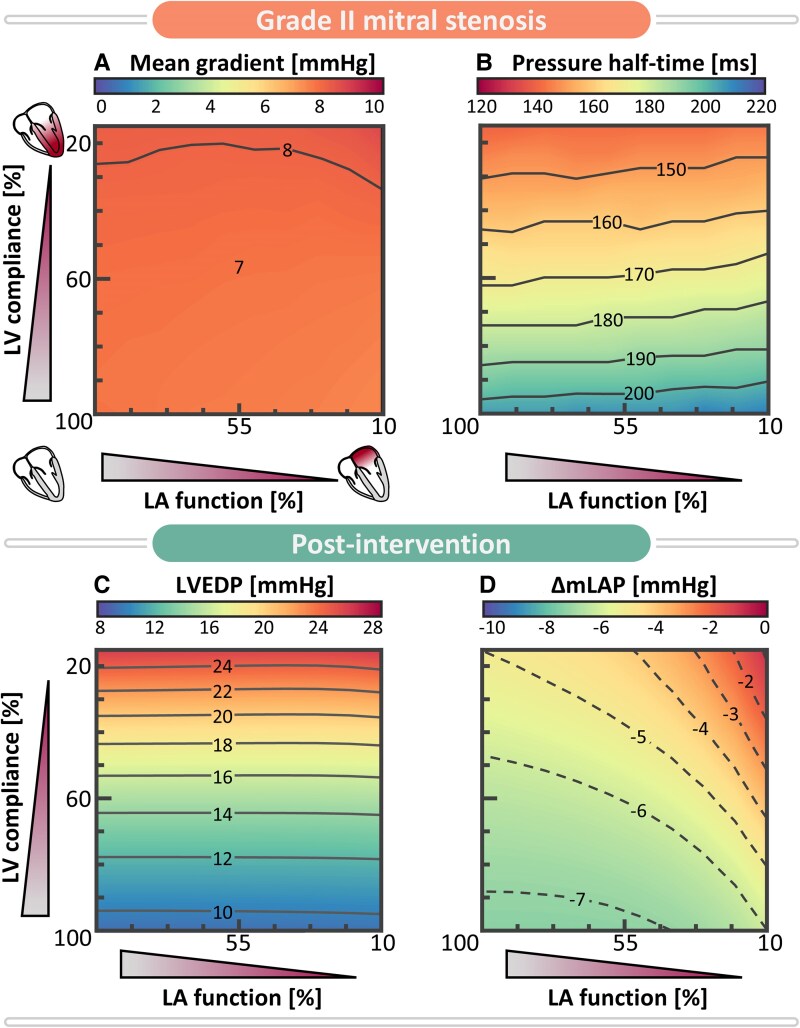
Contour maps indicating the relation between decreasing left atrial (LA) function (left-to-right) and decreasing left ventricular (LV) compliance (bottom-to-top) for pre-intervention non-invasive indices (mean gradient and pressure half-time, top row) and post-intervention outcome (bottom row) in terms of left ventricular end-diastolic pressure (LVEDP) and change of mean left atrial pressure (ΔmLAP) for a Grade II mitral stenosis (MS). Pre- and post-intervention indices for Grades I and III MS are presented in *Supplementary material G*.

*Figure 7* illustrates 100 virtual patients with Grade II MS, presenting with different combinations of reduced LV compliance and impaired LA function. MV intervention was also performed on the virtual cohorts with Grades I and III MS (see *Supplementary material G*). MG remains relatively constant across varying degrees of left-heart myopathy, although variability up to 1.3 mmHg emerges in case of severely reduced LV compliance (*Panel A*). Additionally, PHT is highly sensitive to alterations in LV compliance (*Panel B*). *Panel C* demonstrates that LVEDP remains sensitive to variations in LV compliance after intervention, ranging from 9 to 25 mmHg. Consequently, elevated LVEDP may still be present in the absence of MS, depending on underlying myocardial characteristics. Finally, *Panel D* illustrates mLAP reduction following MV intervention, with the largest decrease of 7 mmHg (50%) observed in virtual patients with healthy myocardial tissue. When LA function is impaired or LV compliance decreased, the effect of intervention reduces to a 6 mmHg (40%) and 5 mmHg (24%) drop respectively. The least notable reduction in mLAP, at 1 mmHg (<4%), is observed in the case of combined LA and LV myopathy. This demonstrates that a reduction in MG by 8 mmHg following MV intervention does not necessarily correspond to a 8 mmHg decrease in mLAP. Consequently, symptoms of congestion may persist despite the MV intervention.

### Acute effect of mitral stenosis and mitral valve intervention on exercise haemodynamics

**Figure 8:**
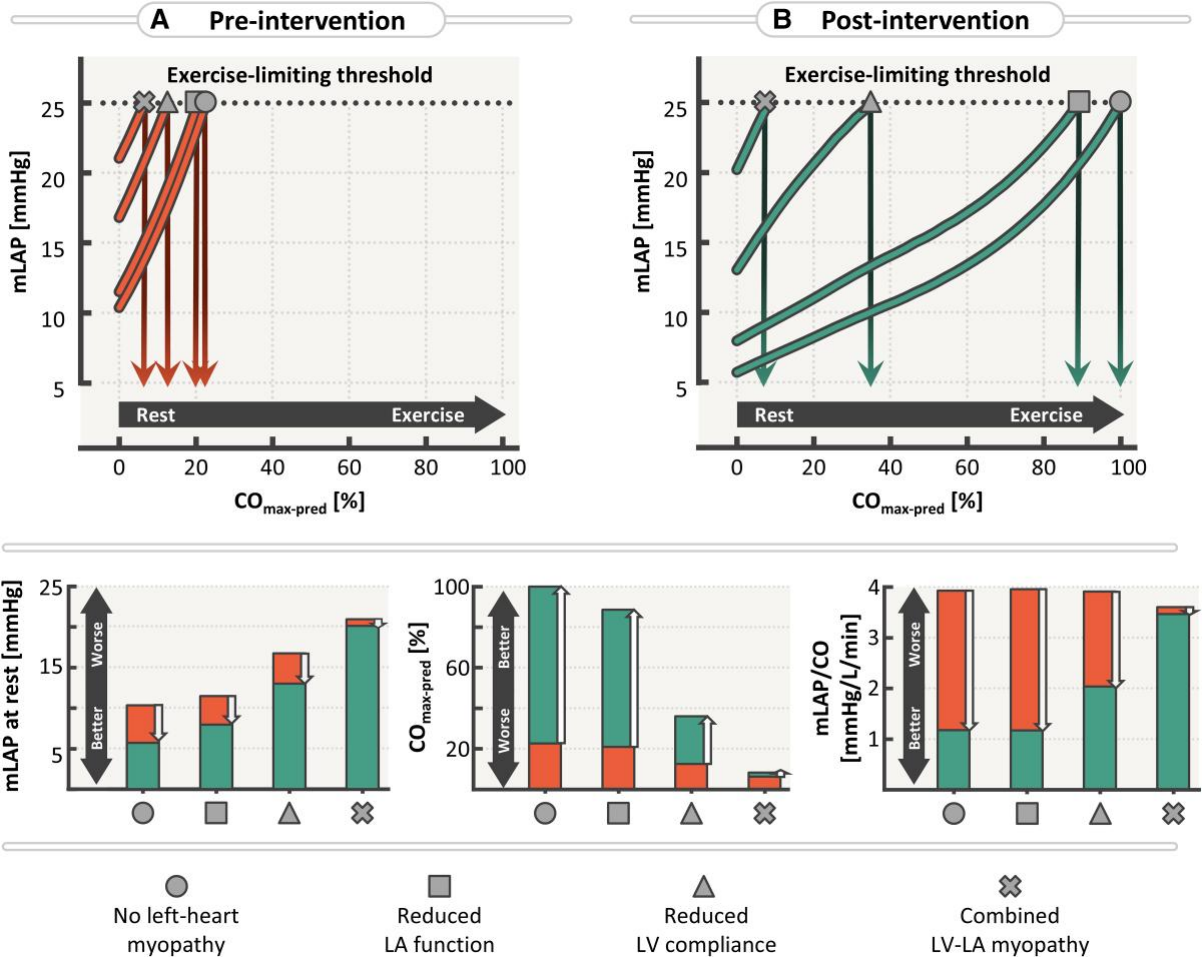
Mean left atrial pressure (mLAP) responses to increasing exercise intensity in four representative virtual patients at the extremes of the myocardial spectrum, shown pre- (Grade II mitral stenosis, Panel A) and post-intervention (Panel B). Arrows in (*A* and *B*) indicate the percentage of predicted maximum cardiac output (CO_max-pred_) at which the exercise-limiting mLAP threshold of 25 mmHg is exceeded. Arrows in the bottom panel represent intervention-induced changes in haemodynamics. LA, left atrium; LV, left ventricle.

*Figure 8* illustrates the dynamic interactions between Grade II MS and left-heart myopathy on mLAP in response to increasing exercise intensity in four representative virtual patients with MS with varying myocardial characteristics.

*Figure 8A* demonstrates that Grade II MS imposes a substantial limitation on exercise capacity, with all virtual patients reaching the pressure threshold well before achieving normal predicted output (CO_max-pred_ < 25%). The addition of LA or LV dysfunction further exacerbates this limitation, with exercise tolerance dropping to 21%, 12%, and just 6% in the presence of isolated LA dysfunction, reduced LV compliance, and combined LV and LA myopathy, respectively.

Following MV intervention (*Figure 6B*), the restoration of exercise capacity varies markedly depending on myocardial characteristics. The virtual patients with normal LV compliance (circle and square) show significant recovery, reaching 100% and 88% of predicted capacity, with mLAP/CO slopes decreasing to 1.17 and 1.16 mmHg/L/min, respectively. In contrast, those with impaired LV compliance (triangle and cross) demonstrate only modest improvement (CO_max-pred_ = 36% and 8%, respectively), despite reductions in resting mLAP with mLAP/CO slopes decreasing to 2.0 and 3.4 mmHg/L/min, respectively. This indicates that while MV intervention alleviates passive obstruction, persistent ventricular stiffness remains a limiting factor during exercise, exacerbated by impaired LA function.

## Discussion

The recent epidemiological transition in the aetiology of MS from predominantly rheumatic to calcific valve disease, often seen in elderly patients with concurrent myocardial impairments (e.g. HFpEF), complicates echocardiographic differentiation between MV disease and cardiomyopathy as the cause of haemodynamic abnormalities and impacts treatment strategies.^7,20–22^ Our virtual patient study demonstrates that: (i) MG is less sensitive to left-heart myopathy compared with PHT, under controlled flow conditions (ii) reductions in MG do not consistently lead to corresponding reductions in mLAP after MV intervention, limiting its utility in predicting haemodynamic benefit post-intervention and (iii) while MV intervention may reduce mLAP at rest, exercise capacity can remain constrained due to underlying myocardial characteristics. This highlights the need for an integrative approach for accurate assessment and management of patients with MS. The results from this study serve as a preliminary step towards clinical validation, providing guidance for future research and clinical practice.

### MG is less sensitive to myocardial disease compared with PHT

MG has previously been demonstrated to correlate well with invasively measured MG^23^ and serves as an important prognostic marker in both rheumatic^20^ and degenerative calcified MV disease.^24^ Our virtual patients demonstrate a non-linear increase in MG as MV EOA decreases, which is consistent with Bernoulli’s law and clinical observations.^9,21^ Furthermore, our findings suggest that MG is a relatively reliable marker for MS severity under constant flow conditions. However, its value can be slightly overestimated in the presence of reduced LV compliance, which increases E-wave flow velocity and, consequently, MG. In this *in-silico* study, the maximum variation attributed to myocardial characteristics was 1.5 mmHg, close to the range of reported inter-observer variability (±1 mmHg).^23^ While this variation is modest, it underscores the potential for minor influence from LV compliance, as previously hypothesized.^6^ While our results are in favour of the utility of MG in accurately classifying MS severity in the presence of left-heart myopathy, we acknowledge its inherent dependence on flow. Factors like concomitant mitral regurgitation, high-flow states (e.g. anaemia, hyperthyroidism, and sepsis), low-flow conditions (e.g. severe MS, bradycardia, and atrial fibrillation), and tachycardia often complicate the clinical interpretation of MG. Our results confirmed this dependency on transmitral flow and further demonstrated that variability in MG due to myocardial characteristics becomes more pronounced as transvalvular flow increases. Therefore, a comprehensive evaluation of stenosis severity based on MG should include HR and, ideally, CO, diastolic function and a careful evaluation of mitral regurgitation, particularly within the context of mixed valvular disease.

In contrast, PHT demonstrated greater sensitivity to myocardial abnormalities, particularly LV compliance. While PHT-derived MVA using the Hatle formula remains widely adopted for assessing rheumatic MS,^7,20^ our findings align with prior literature highlighting its limitations in cases of reduced LV compliance where PHT tends to underestimate MS severity.^20,25^ Interestingly, impaired LA function, which can be common in long-standing rheumatic MS, appeared to have little impact on the PHT-derived MVA calculation, as long as the LV compliance remains normal. These findings further highlight the limitations of PHT in the context of myocardial dysfunction and reinforce the relative robustness of MG as a diagnostic marker under controlled flow conditions.

### Elevated filling pressures result from the interplay between mitral stenosis and myocardial function

The reported variation in outcomes after MV intervention emphasizes the absence of a consistent, direct correlation between the MG and mLAP.^26–28^ This is confirmed by a study of Eleid *et al*.^26^ in which a cohort of patients with severe MS was sub-divided into normal and increased filling pressures (defined as LVEDP > 15 mmHg). Both CO and MG values at baseline were not significantly different before percutaneous mitral balloon valvotomy (PMBV). Following PMBV, MG decreased in both groups, while mean pulmonary arterial pressure and mLAP remained high in the increased LVEDP group. Additionally, symptomatic improvement was significantly less in the increased filling pressure group compared with the normal filling pressure group. These findings align with our simulations, implying that MG is specific for MS severity, regardless of myocardial characteristics, and can be addressed with MV intervention. However, a reduction in MG does not necessarily result in a corresponding decrease in mLAP post-intervention, as this outcome also depends on underlying myocardial factors. Our simulations suggest that reduced LV compliance, whether alone or combined with impaired LA function, can result in persistently elevated LVEDP and mLAP, even after MV intervention. This underscores the importance of considering LVEDP in diagnostics, as it may be an important predictor of intervention outcome, particularly in the elderly with degenerative calcified MV disease.

### Myocardial characteristics determine exercise capacity after mitral valve intervention

While resting indices for valvular and myocardial function provide valuable diagnostic and prognostic insights, our virtual exercise protocol reveals that myocardial characteristics, particularly LV compliance combined with impaired LA function, are critical determinants of exercise capacity. Our findings suggest that even after successful MV intervention with reduction of MG and mLAP, patients with impaired LV compliance and LA function may continue to experience exertional symptoms as their cardiovascular system is unable to meet circulatory demands.

Chronic valve disease can induce structural myocardial changes that may persist despite correction of the valvular lesion and is often progressive and irreversible. Our findings lend support to considering early intervention in cases where remodelling is primarily driven by the valvular pathology, possibly before symptom onset, to prevent advanced LV and/or LA remodelling. However, clinical evidence supporting early intervention remains limited.^29–31^

### Clinical implications

The current study suggests that solely targeting the MG should be avoided and emphasizes the importance of differentiating between elevation of filling pressure caused by valvular disease vs. myocardial disease. This distinction is critical given the increasing use of percutaneous options for valvular treatment in HFpEF patients, where reducing MG may not necessarily decrease mLAP or lead to an increase in exercise capacity. According to our simulations, quantifying LVEDP is essential because (i) LVEDP is not directly influenced by MS severity, and (ii) elevated LVEDP can result in persistently high mLAP following intervention, preventing symptomatic relief at rest and during exercise. However, non-invasive assessment of LV diastolic pressures remains challenging, even more so in the context of degenerative MS and HFpEF.^32–34^ Our virtual cohort study hypothesizes that overestimations of MVA derived from PHT, as compared with MG-based MS severity assessment, may indicate underlying reduced LV compliance, contributing to elevated LVEDP. Future *in vivo* studies should, therefore, investigate the diagnostic implications of discrepancies between flow velocity-based echocardiographic indices for MS severity and left-heart myopathy within individual patients to establish clinical relevance.

Additionally, advancements in personalized computational modelling techniques, such as digital twin technology, may offer opportunities to bridge the existing gap in understanding the cardiovascular pathology underlying patients’ clinical symptoms, thereby supporting patient-specific clinical decision-making and improving patient selection for MV intervention. Importantly, such haemodynamic assessment should be interpreted as complementary to anatomical evaluation, which remains fundamental for comprehensive clinical assessment and decision-making in patients with MS.

### Limitations

All virtual cohorts were generated assuming a constant CO of 5.1 L/min with a HR of 70 beats per minute at rest, regardless of MS severity, myocardial function, or MV intervention. This approach allowed us to investigate the modulating effect of MS severity and left-heart myopathy independently of flow and rate variabilities. Current guidelines indeed emphasize the inherent dependency of the MG on transmitral flow,^7^ which is particularly important in conditions such as tachycardia, anaemia, atrial fibrillation, and concomitant mitral regurgitation. In line with the guidelines, our virtual cohort demonstrated misclassification of MS severity based on MG when subjected to varying preload conditions (*Figure 5*).

Additionally, impaired LA function in this study represents the acute effect of reduced atrial contractility and volume overload. It does not incorporate long-term structural remodelling, such as fibrosis or eccentric hypertrophy, which may further impair atrial function and haemodynamics. Similarly, our simulations do not incorporate mechanical effects during balloon mitral valvotomy other than a variation in MV EOA, which may acutely alter the mitral apparatus and lead to immediate improvements in LV function, as have been observed clinically.^35,36^

Moreover, our model does not differentiate between aetiology-specific valve haemodynamics but instead focuses on the functional obstruction resulting from the modelled effective orifice area.

Furthermore, we acknowledge that our model and mechanistic approach do not fully capture the complexity of the comorbid population, notably excluding the complex 3D morphology of the MV as well as pulmonary vascular and right ventricular function. Nonetheless, our findings from this well-controlled and systematic approach provide valuable insights into the intricate interactions between the atrium, ventricle, and valve. These insights are, by nature, hypothesis-generating and may guide future research towards *in vivo* validation. Such validation will be crucial to confirm the results of this study and translate them into clinical practice, potentially improving patient care and treatment strategies.

## Conclusions

Our virtual patient cohort suggests that MG is less sensitive to left-heart myopathy compared with PHT, assuming known flow conditions. Additionally, our *in-silico* trial implies that while MV intervention decreases MG, persistently elevated LVEDP can continue to elevate LA pressure in hearts with impaired LV compliance and LA function, potentially limiting symptomatic relief following intervention. Importantly, exercise simulations reveal that these myocardial characteristics impair the ability to meet circulatory demands during stress, highlighting exercise intolerance as a key residual burden even after technically successful MV intervention. Given the rise of minimally invasive, percutaneous treatments for MV disease, our study emphasizes the importance of the ability to differentiate between valve- or myocardial-related causes of haemodynamic abnormalities.

## Supplementary Material

ztaf097_Supplementary_Data

## Data Availability

The data underlying this article will be shared on reasonable request to the corresponding author.
