# Supplementary material for: Impact of left-heart myopathy on mitral valve stenosis assessment and interventional outcomes: an in-silico trial
Source: Eur Heart J Digit Health. 2025 Aug 19;7(1):ztaf097. doi: 10.1093/ehjdh/ztaf097 (PMC12821063; doi:10.1093/ehjdh/ztaf097)
Supplement: ztaf097_Supplementary_Data [file ztaf097_supplementary_data.docx]

**Supplementary materials**

Impact of Left-Heart Myopathy on Mitral Stenosis Assessment and Interventional Outcomes: An in-silico trial

**Supplementary material contents**

[Supplementary material A: The CircAdapt valve module 2](#_Toc198841053)

[Supplementary material B: Derivation of simplified Bernoulli equation 3](#_Toc198841054)

[Supplementary material C: Calculating pressure half-time 3](#_Toc198841055)

[Supplementary material D: Calculating left atrial stiffness 3](#_Toc198841056)

[Supplementary material E: Calculating left ventricular diastolic chamber stiffness 4](#_Toc198841057)

[Supplementary material F: Modulating effect of heart rate on (non-)invasive indices 5](#_Toc198841058)

[Supplementary material G: Acute intervention results for mild and severe mitral stenosis 6](#_Toc198841059)

[References 7](#_Toc198841060)

## Supplementary material A: The CircAdapt valve module

The mitral valve (MV) is modelled as previously described [1, 2] and connects the proximal (left atrium, LA) and distal (left ventricle, LV) cavities with a small cylindrical channel with corresponding cross-sectional areas $A_{LA}$, $A_{MV}$and $A_{LV}$ (*Figure* **S1**). The passing blood flow causes a pressure drop related to the effects of inertia and Bernoulli and can be described using the Navier-Stokes equation, assuming an incompressible fluid:

$\nabla p+ \rho_{b}\frac{d\boldsymbol{v}}{dt}+ \rho_{b}\left( \boldsymbol{v}\cdot\nabla\right)\mathbf{v}- \eta\nabla\cdot\nabla\mathbf{v}=0$ (S1)

where $\rho_{b}$ and $\eta$ refer to blood density and viscosity, respectively. When assuming laminar flow with a dominance in axial direction, the pressure gradient ($\Delta p = p_{LA} - p_{LV}$) follows from integration along a streamline, neglecting viscosity and the effect of gravity over the short distance within the valve:

$\Delta p= {\rho_{b}l}_{MV}\frac{{dv}_{MV}}{dt}+\frac{1}{2}\rho_{b}\left( v_{LV}-v_{LA} \right)^{2}$ (S2)

in which $l_{MV}$ refers to the length of the MV, $\frac{{dv}_{z}}{dt}$ to the change in blood flow velocity in time and $v_{LV}$and $v_{LA}$represent the blood flow velocities at the LV and LA. The divergence of streamlines distal to the orifice leads to vortices and turbulence with loss of energy. To account for this, we assumed that there is no pressure regained by deceleration of the blood distal to the orifice and therefore, energy is lost. *Eq.* ***S2*** can then be rewritten as follows:

$\Delta p= {\rho_{b}l}_{MV}\frac{{dv}_{MV}}{dt}+\frac{1}{2}\rho_{b}\left\{ \begin{aligned} v_{max}^{2}\left( t \right)-v_{LA}^{2}\left( t \right) \mathrm{if} q_{MV}\geq0 \\ v_{LV}^{2}\left( t \right)-v_{max}^{2}\left( t \right)\mathrm{if} q_{MV}<0 \end{aligned} \right.$ (S3)

$$with v_{max}^{2}=\max\left( v_{LA}^{2}, v_{MV}^{2}, v_{LV}^{2} \right)$$

flow velocities $v_{LA}$, $v_{MV}$and $v_{LV}$are determined by dividing the flow $q_{mv}(t)$ by the cross-sectional area $A_{LA}$, $A_{MV}$and $A_{LV}$, respectively. The effective orifice area (EOA) of the MV at time t is determined by the pressure gradient and flow. The MV opens when the pressure gradient is positive $(p_{LA}\left( t \right)>p_{LV}\left( t \right))$, allowing forward flow from the LA into the LV. In this case, EOA is set to 5.9 cm^2^. When $p_{LA}\left( t \right)=p_{LV}(t)$, the MV starts closing and while the pressure gradient becomes negative $(p_{LA}\left( t \right)<p_{LV}\left( t \right))$, forward flow briefly remains due to inertial effects. The MV fully closes when the pressure gradient is negative, and no forward flow remains.


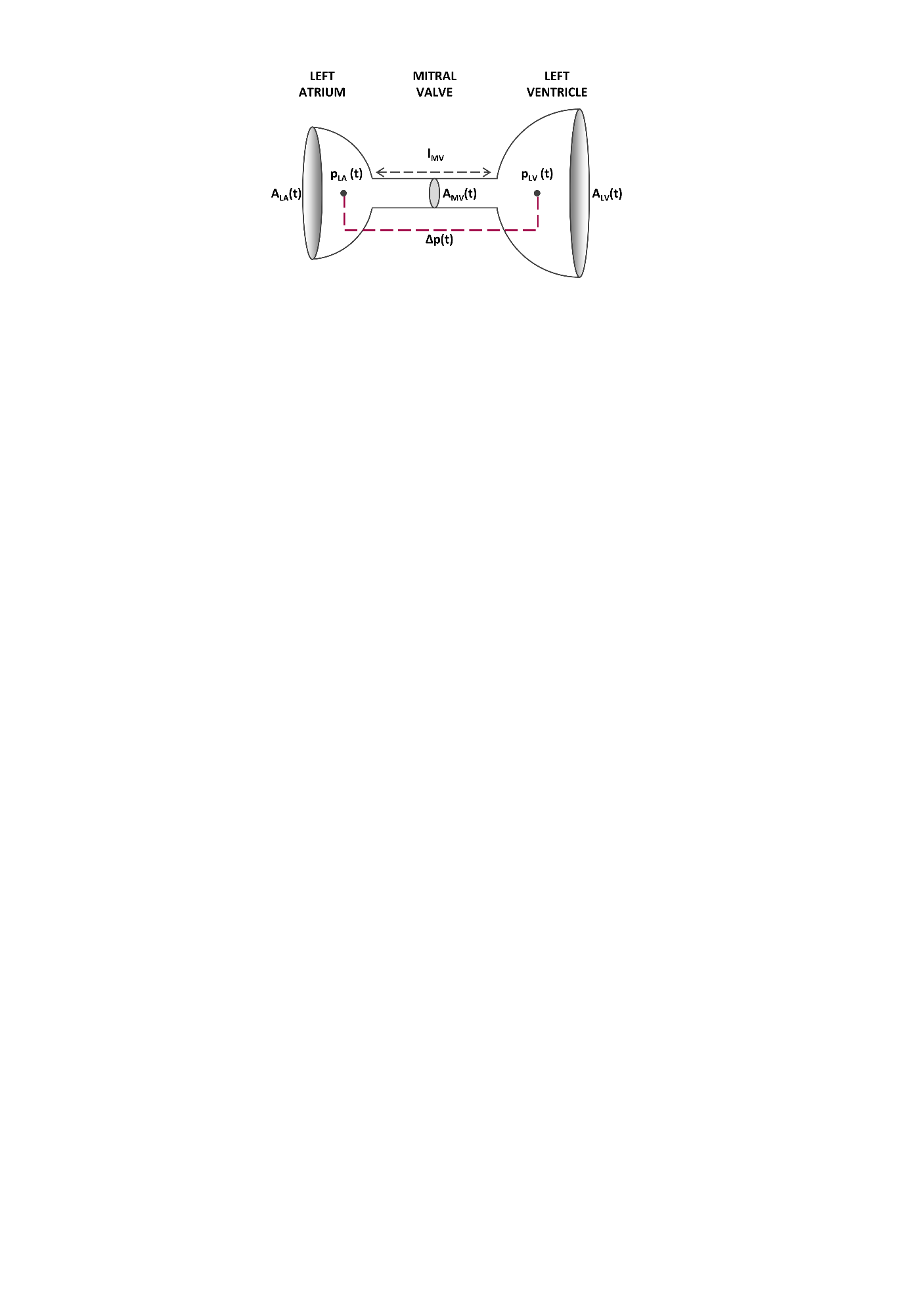


**Figure S1.** Schematic representation of the mitral valve (MV). Symbols A, l and p represent cross-sectional area, length and pressure, respectively, for the left ventricle (LV), left atrium (LA) and MV at time t. Δp reflects the transmitral pressure gradient at time t.

## Supplementary material B: Derivation of simplified Bernoulli equation

Assuming unsteady, incompressible and non-viscous flow, and neglecting the effect of gravity, the Bernoulli equation for unsteady flow applied to the blood flow in the left atrium (LA) and mitral valve (MV) is as follows:

$\rho_{b}l_{MV}\cdot\frac{dv_{MV}}{dt}+\frac{1}{2}\rho_{b}\left( v_{MV}^{2}-v_{LA}^{2} \right)+\left( p_{MV}-p_{LA} \right)=0$ (S4)

When neglecting the inertial term, *Eq.* ***S4*** can be rewritten as:

$\frac{1}{2}\rho_{b}\left( v_{MV}^{2}-v_{LA}^{2} \right)= p_{LA}-p_{MV}$ (S5)

Assuming that there is no pressure difference between the MV and left ventricle (LV) and that the flow velocity in the LA is much smaller than the flow velocity in the MV $(v_{LA}<<v_{MV})$, the simplified Bernoulli equation can be derived:

$\frac{1}{2}\rho_{b}v^{2}=4v^{2}=\Delta p$ (S6)

where $v$ represents the flow velocity over the MV, and $\Delta p$ is the pressure gradient across the MV. Note that $\frac{1}{2}\rho$ can be approximated by 4 when the pressure gradient is expressed in mmHg.

## Supplementary material C: Calculating pressure half-time

The pressure half-time (PHT) is defined as the time required for the maximum transvalvular pressure gradient $(\Delta p_{max})$ to decay to half of its value, so:

$\Delta p_{PHT}= \frac{\Delta p_{max}}{2}$ (S7)

In the clinical setting, PHT is computed as the time for the maximum flow velocity, $v_{max}$, to decay to $v_{PHT}$ whose value is such that $\Delta p_{PHT}= \frac{1}{2}\rho v_{PHT}^{2}$ . $v_{PHT}$can be calculated using the simplified Bernoulli expression (*Eq.* ***S6***).

$\Delta p_{PHT}= \frac{1}{2}\rho_{b}v_{PHT}^{2}{\to v}_{PHT}=\sqrt{\frac{2\Delta p_{PHT}}{\rho_{b}}} \underset{\to}{Eq. S7} v_{PHT}=\sqrt{\frac{\Delta p_{max}}{\rho_{b}}} \underset{\to}{Eq. S6} v_{PHT}=\frac{v_{max}}{\sqrt{2}}$ (S8)

## Supplementary material D: Calculating left atrial stiffness

Operant left atrial (LA) stiffness was characterized following the method used by Melenovsky et al. (2015) [3] which involves taking the slope of the linear regression (α), connecting the minimum and maximum LA pressures during reservoir phase to their corresponding volumes (*Figure* **S2***, reference*). To ensure only the reservoir phase was considered, the minimum LA pressure was determined at aortic valve opening and the maximum LA pressure at mitral valve opening [4].

**
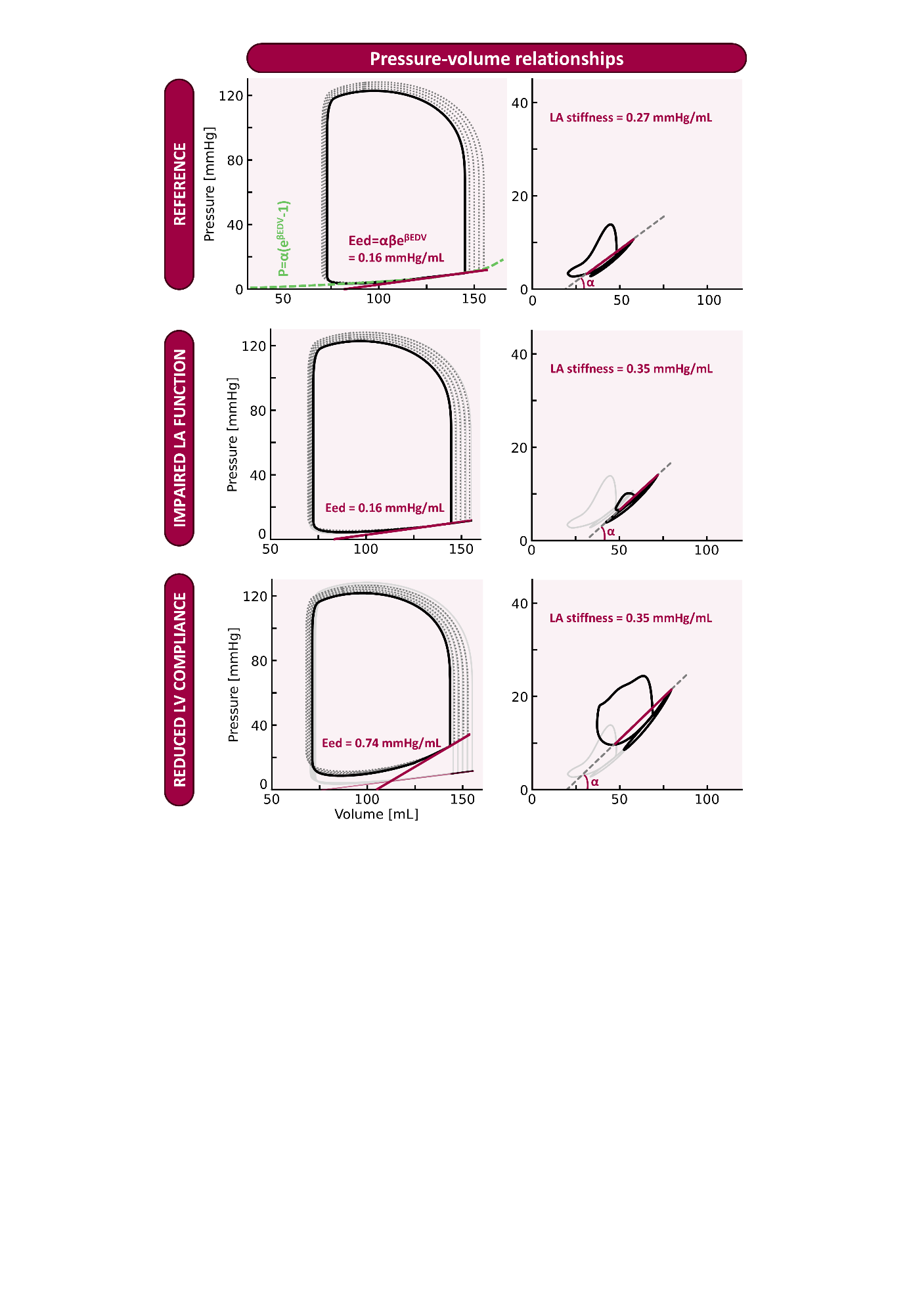
**

**Figure S2.** Pressure-volume loops of the left ventricle **(left column)** and left atrium **(right column)** for different left-heart myopathies. For the left ventricle, an in silico­ preload experiment was performed **(gray dotted lines)** to obtain the end-diastolic pressure volume relation, from which the end-diastolic elastance (Eed) was derived. For the left atrium, operant stiffness was determined by taking the slope of the line connecting the minimum and maximum LA pressure-volume points.

## Supplementary material E: Calculating left ventricular diastolic chamber stiffness

The left ventricular (LV) diastolic chamber stiffness was calculated as was established by Burckhoff et al. (2005) [5] and previously applied to the CircAdapt model by Van Loon et al. (2020) [6]. This involved determining the end-diastolic elastance (Eed) by fitting the non-exponential curve through the end-diastolic LV volume point of the pressure-volume loops (*Figure* **S2***, reference*). The exponential curve is given by:

$P= \alpha\left( e^{\beta EDV}-1 \right)$ (S9)

where $P$ is the LV pressure, $\alpha$ and$\beta$ curve fitting coefficients and $EDV$ the end-diastolic volume of the LV at different loading conditions. In the CircAdapt model, these different loading conditions were achieved by incrementally increasing the cardiac output from 5.1 L/min (black line) to 6.1 L/min in steps of 0.1 L/min (grey dotted line). Finally, Eed was determined by taking the slope of the end-diastolic pressure volume relation as follows:

$Eed= \frac{dP}{dV}=\alpha\beta e^{\beta EDV}$ (S10)

## Supplementary material F: Modulating effect of heart rate on (non-)invasive indices

In the CircAdapt model, cardiac output (CO) can be tightly regulated at a set value for a given heart rate (HR) and mean arterial pressure, effectively simulating homeostatic regulation. The model adheres to the relationship CO = HR x stroke volume (SV), ensuring that any changes in HR are counterbalanced by proportional adjustments in SV, thereby maintaining the target CO. This dynamic reflects compensatory mechanisms observed in vivo.

The impact of variability in HR on non-invasive diagnostics, such as mean gradient (MG) and pressure half-time (PHT), as well as on left-heart pressures – specifically left ventricular end-diastolic pressure (LVEDP) and mean left atrial pressure (mLAP) – was evaluated under the assumption of constant systemic flow (i.e. CO), mean arterial pressure (MAP), and HR, in case of a moderate mitral stenosis.

As shown in Figure **S3**, pressure half-time (PHT) demonstrates minimal sensitivity to heart rate variations, whereas the mean gradient (MG) exhibits greater sensitivity. This is attributable to the model’s flow regulation, where any change in HR is offset by a corresponding adjustment in SV, thereby preserving the average transvalvular flow during the diastolic time. For example, a reduced heart rate increases stroke volume, which, particularly in the presence of a stiffened left ventricle, elevates instantaneous flow velocities and consequently leads to an overestimation of the mean gradient. To assess the potential impact of HR-induced changes in CO on indices for mitral stenosis assessment, additional simulations were performed at different CO, as shown in *Figure 5* in the main manuscript.

*Figure* **S3** additionally shows that an increase in SV due to a decrease in HR is generally well tolerated in terms of mLAP and LVEDP, provided that there is no left-heart myopathy or only isolated impairment of left atrial (LA) function. However, in cases of reduced left ventricular (LV) compliance, the resultant increase in SV leads to a significant rise in mLAP. This sensitivity is due to the increased non-linearity of the passive stress-strain relationship in the stiffened LV myocardium, where even minor volume changes lead to substantial pressure elevations.


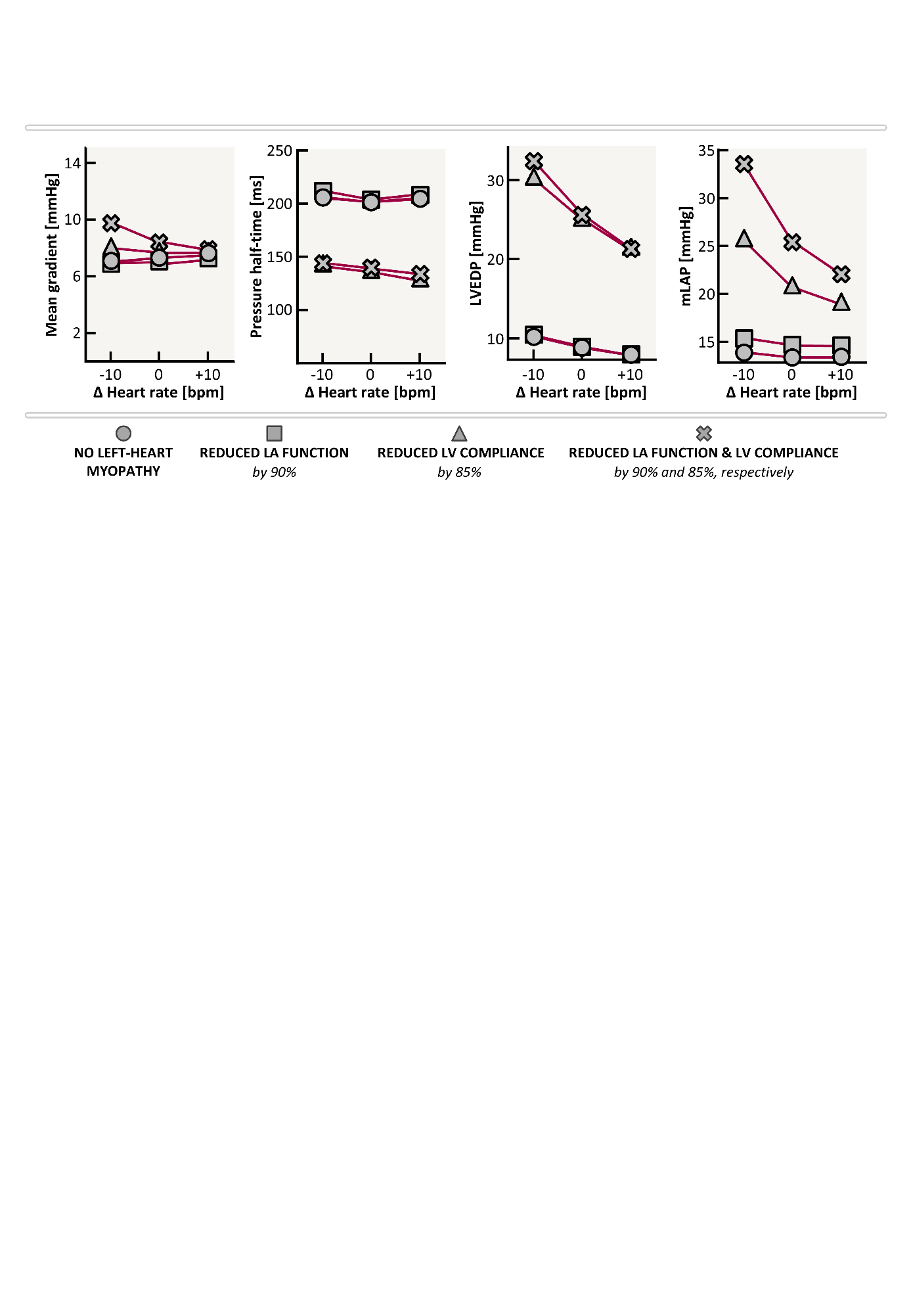


**Figure S3.** Effect of variability in heart rate (HR) on the non-invasive indices for mitral stenosis (MS) severity assessment and left-heart pressures in the presence and absence of left-heart myopathy. Symbols represent myopathy types: circle (no left-heart myopathy), square (impaired left atrial (LA) function), triangle (reduced left ventricular (LV) compliance), and cross (reduced LV compliance with impaired LA function). Indices for MS severity assessment include the mean gradient and pressure half-time, while left ventricular end-diastolic pressure (LVEDP) and mean left atrial pressure (mLAP) represent the left-heart pressures. HR was changed with respect to the baseline model setting of 70 beats per minute (bpm).

## Supplementary material G: Acute intervention results for mild and severe mitral stenosis


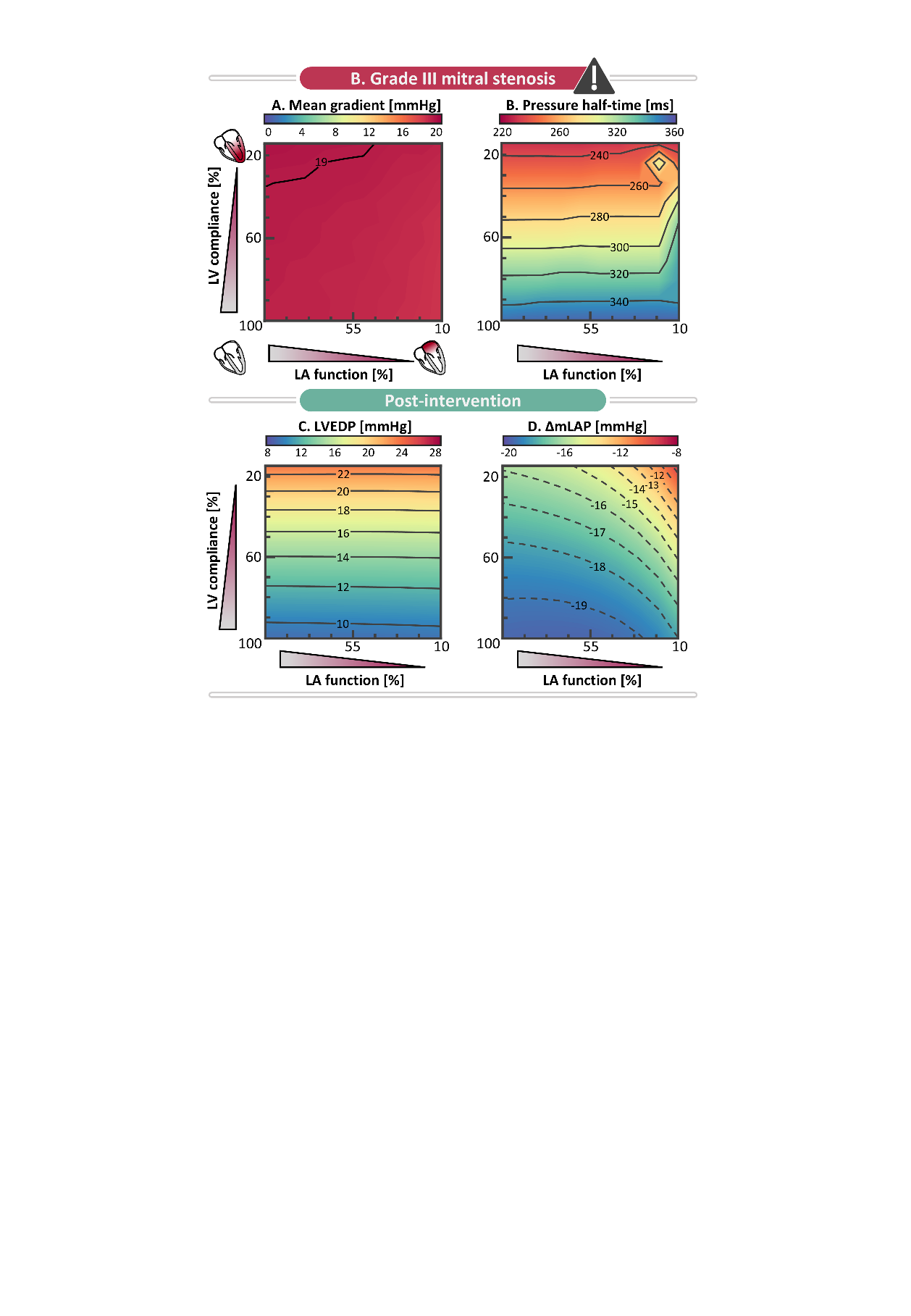
The acute effects of mitral valve (MV) intervention were simulated for the virtual patient cohort, considering grades I and III of mitral stenosis (MS) severity. Mean gradient shows modest sensitivity in cases of extreme myocardial abnormalities. The pressure half-time in both cases shows sensitivity towards reduced left ventricular (LV) compliance. End-diastolic pressures remain elevated following intervention in cases of reduced LV compliance. Finally, the absolute decrease in mean left atrial pressure following MV intervention depends not only on MS severity, but also on the underlying myocardial characteristics, with a limited reduction in mLAP in case of combined LV and left atrial myopathy.


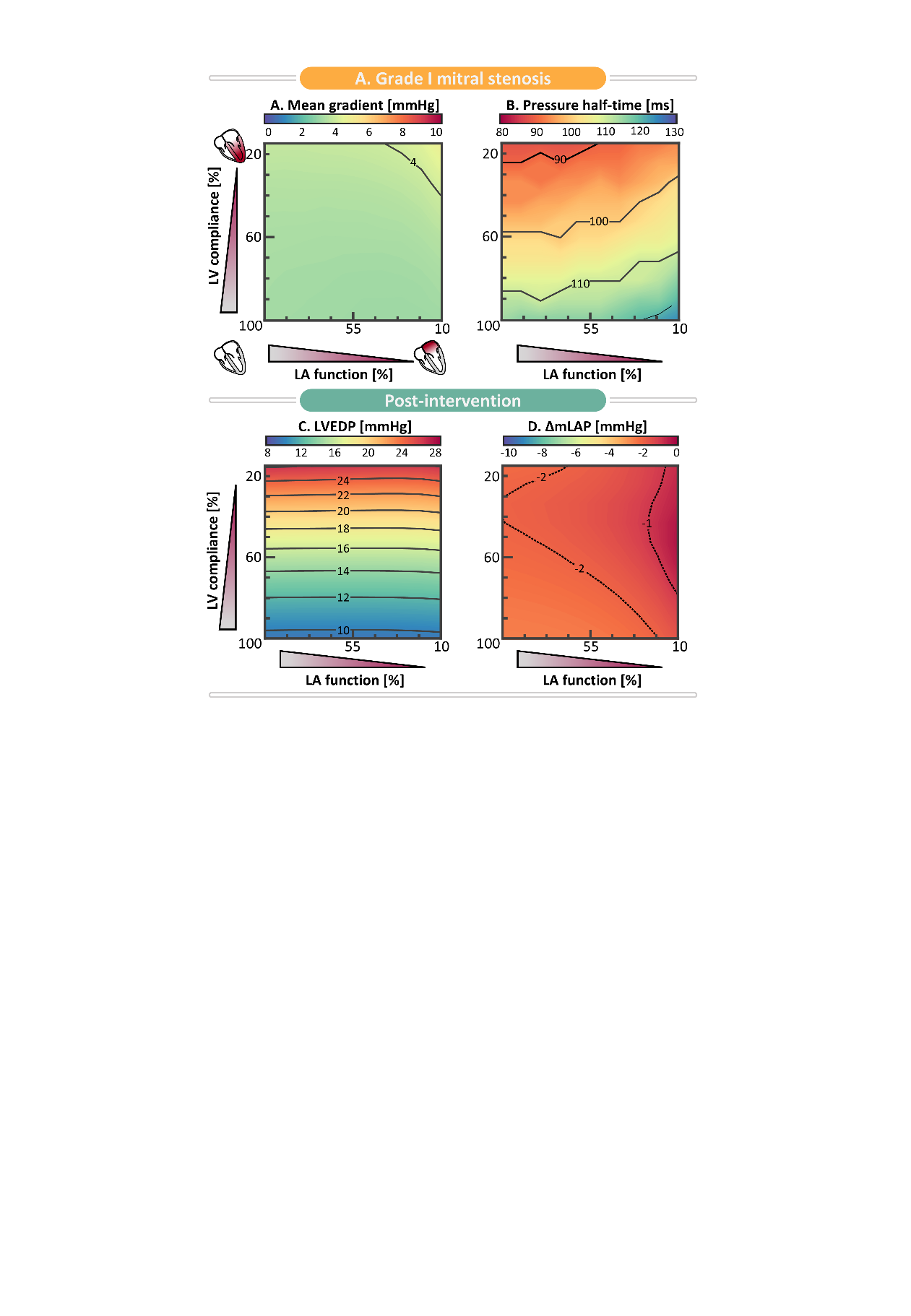


**Figure S4.** Contour maps indicating the relation between decreasing left atrial (LA) function (left-to-right) and decreasing left ventricular (LV) compliance (bottom-to-top) for pre-intervention non-invasive indices (mean gradient and pressure half-time, top row) and post-intervention outcome (bottom row) in terms of left ventricular end-diastolic pressure (LVEDP) and change of mean left atrial pressure (ΔmLAP) for a mild mitral stenosis (MS) **(*Panel A*)** and severe MS **(*Panel B*)** according to guidelines for valvular disease [7].

## References

[1] T. Arts, T. Delhaas, P. Bovendeerd, X. Verbeek, and F. W. Prinzen, "Adaptation to mechanical load determines shape and properties of heart and circulation: the CircAdapt model," *American Journal of Physiology-Heart and Circulatory Physiology,* vol. 288, no. 4, pp. H1943-H1954, 2005, doi: 10.1152/ajpheart.00444.2004.

[2] J. Lumens, T. Delhaas, B. Kirn, and T. Arts, "Three-Wall Segment (TriSeg) Model Describing Mechanics and Hemodynamics of Ventricular Interaction," *Annals of Biomedical Engineering,* vol. 37, no. 11, pp. 2234-2255, 2009/11/01 2009, doi: 10.1007/s10439-009-9774-2.

[3] V. Melenovsky, S.-J. Hwang, M. M. Redfield, R. Zakeri, G. Lin, and B. A. Borlaug, "Left Atrial Remodeling and Function in Advanced Heart Failure With Preserved or Reduced Ejection Fraction," *Circulation: Heart Failure,* vol. 8, no. 2, pp. 295-303, 2015, doi: doi:10.1161/CIRCHEARTFAILURE.114.001667.

[4] S. Dhont *et al.*, "Mitral regurgitation in heart failure with preserved ejection fraction: The interplay of valve, ventricle, and atrium," *European Journal of Heart Failure,* vol. 26, no. 4, pp. 974-983, 2024, doi: <https://doi.org/10.1002/ejhf.3231>.

[5] D. Burkhoff, I. Mirsky, and H. Suga, "Assessment of systolic and diastolic ventricular properties via pressure-volume analysis: a guide for clinical, translational, and basic researchers," *American Journal of Physiology-Heart and Circulatory Physiology,* vol. 289, no. 2, pp. H501-H512, 2005, doi: 10.1152/ajpheart.00138.2005.

[6] T. van Loon *et al.*, "Increased myocardial stiffness more than impaired relaxation function limits cardiac performance during exercise in heart failure with preserved ejection fraction: a virtual patient study," *European Heart Journal - Digital Health,* vol. 1, no. 1, pp. 40-50, 2020, doi: 10.1093/ehjdh/ztaa009.

[7] A. Vahanian *et al.*, "2021 ESC/EACTS Guidelines for the management of valvular heart disease: Developed by the Task Force for the management of valvular heart disease of the European Society of Cardiology (ESC) and the European Association for Cardio-Thoracic Surgery (EACTS)," *European Heart Journal,* vol. 43, no. 7, pp. 561-632, 2021, doi: 10.1093/eurheartj/ehab395.
